# Rising Diabetes Prevalence among Urban-Dwelling Black South Africans

**DOI:** 10.1371/journal.pone.0043336

**Published:** 2012-09-04

**Authors:** Nasheeta Peer, Krisela Steyn, Carl Lombard, Estelle V. Lambert, Bavanisha Vythilingum, Naomi S. Levitt

**Affiliations:** 1 Chronic Diseases of Lifestyle Unit, Medical Research Council, Durban, South Africa; 2 Chronic Disease Initiative in Africa, Department of Medicine, University of Cape Town, Cape Town, South Africa; 3 Biostatistics Unit, Medical Research Council, Cape Town, South Africa; 4 University of Cape Town/Medical Research Council Research Unit for Exercise Science and Sports Medicine, Cape Town, South Africa; 5 Department of Psychiatry, University of Cape Town, Cape Town, South Africa; 6 Division of Endocrinology and Diabetes, Department of Medicine, University of Cape Town, Cape Town, South Africa; German Diabetes Center, Leibniz Center for Diabetes Research at Heinrich Heine University Duesseldorf, Germany

## Abstract

**Objective:**

To examine the prevalence of and the association of psychosocial risk factors with diabetes in 25–74-year-old black Africans in Cape Town in 2008/09 and to compare the prevalence with a 1990 study.

**Research Design and Methods:**

A randomly selected cross-sectional sample had oral glucose tolerance tests. The prevalence of diabetes (1998 WHO criteria), other cardiovascular risk factors and psychosocial measures, including sense of coherence (SOC), locus of control and adverse life events, were determined. The comparison of diabetes prevalence between this and a 1990 study used the 1985 WHO diabetes criteria.

**Results:**

There were 1099 participants, 392 men and 707 women (response rate 86%). The age-standardised (SEGI) prevalence of diabetes was 13.1% (95% confidence interval (CI) 11.0–15.1), impaired glucose tolerance (IGT) 11.2% (9.2–13.1) and impaired fasting glycaemia 1.2% (0.6–1.9). Diabetes prevalence peaked in 65–74-year-olds (38.6%). Among diabetic participants, 57.9% were known and 38.6% treated. Using 1985 WHO criteria, age-standardised diabetes prevalence was higher by 53% in 2008/09 (12.2% (10.2–14.2)) compared to 1990 (8.0% (5.8–10.3)) and IGT by 67% (2008/09: 11.7% (9.8–13.7); 1990: 7.0% (4.9–9.1)). In women, older age (OR: 1.05, 95%CI: 1.03–1.08, p<0.001), diabetes family history (OR: 3.13, 95%CI: 1.92–5.12, p<0.001), higher BMI (OR: 1.44, 95%CI: 1.20–1.82, p = 0.001), better quality housing (OR: 2.08, 95%CI: 1.01–3.04, p = 0.047) and a lower SOC score (≤40) was positively associated with diabetes (OR: 2.57, 95%CI: 1.37–4.80, p = 0.003). Diabetes was not associated with the other psychosocial measures in women or with any psychosocial measure in men. Only older age (OR: 1.05, 95%CI: 1.02–1.08, p = 0.002) and higher BMI (OR: 1.10, 95%CI: 1.04–1.18, p = 0.003) were significantly associated with diabetes in men.

**Conclusions:**

The current high prevalence of diabetes in urban-dwelling South Africans, and the likelihood of further rises given the high rates of IGT and obesity, is concerning. Multi-facetted diabetes prevention strategies are essential to address this burden.

## Introduction

Diabetes has globally emerged as a major public health challenge of the 21^st^ century. Sub-Saharan Africa (SSA), where diabetes was considered rare prior to the 1990s, has also witnessed a rise in the burden [1], [2]. Studies conducted 10–20 years ago found that diabetes prevalence varied across the region. There are, however, limited data as to the extent of the rise. In urban Tanzania rates increased from 0.3% in the 1980s to 4.6% in 1996 according to the 1998 WHO criteria, and in urban Cameroon from 1.5% in the 1990s to 6.6% in 2003 [1], [2].

Diabetes was associated with 4.3% of South African adult mortality in 2000, placing it among the top ten leading causes of adult deaths in the country at that stage. Approximately 14% of ischaemic heart disease (IHD), 10% of stroke, 12% of hypertensive disease and 12% of renal disease mortality in those ≥30 years were attributable to diabetes [3].

There is a lack of recent data on the prevalence of diabetes in urban South Africans, which constitutes the majority of the population. The last diabetes prevalence study conducted in the urban black African population of Cape Town was in 1990 [4]. Following the trend in other SSA countries the prevalence of diabetes is expected to have risen due to a change in demography with an ageing population, urbanisation, and the increasing prevalence of obesity and physical inactivity [1], [5].

In addition to the traditional risk factors for diabetes, it has been postulated that psychosocial factors may also be associated with diabetes. Studies from developed country settings have demonstrated the association of various psychosocial stressors with diabetes, including adverse life events [6] and a low sense of coherence (SOC) [7], but little is known about the relationship between psychosocial factors and diabetes in SSA.

The current study aimed to: 1) ascertain the prevalence of, and associations with, diabetes in adults living in predominantly black residential areas of Cape Town and to compare these findings with the 1990 study; 2) examine the association between diabetes and psychosocial stress using selected validated questionnaires.

## Materials and Methods

### Study population and sampling procedure

The target population was 25–74-year-old residents living in the predominantly black African areas of Langa, Guguletu, Crossroads, Nyanga and Khayelitsha in Cape Town to provide some overlap with the surveys conducted in 1990. A sample size of 1000 was planned based on an estimated diabetes prevalence of 8% with a precision of 1.5% two-sided with 95% confidence.

Using the 2001 census and aerial maps from 2007, a 3-stage cluster sampling stratified by area and housing type was undertaken as follows: stage 1) random sampling of residential blocks within the main strata; stage 2) systematic sampling of plots, flats or structures within blocks; stage 3) individuals from households were selected using quotas for pre-specified age and gender categories. Sampling across the areas and age groups were disproportionate. Langa was oversampled to accommodate a secondary study. Younger age groups were under-sampled and older age groups were over-sampled to ensure at least 50 men and women in each gender category.

Participants were excluded on the following basis: unable to give consent, on tuberculosis treatment, on antiretroviral therapy, received cancer treatment within the last year, bedridden, pregnant or lactating, or resident in Cape Town for less than 3 months. Replacements were allowed when individuals who met the inclusion criteria refused, or the randomly selected participant of the randomly selected household could not be contacted on the third attempt.

### Data collection

Fieldworkers administered questionnaires to obtain socio-demographic and migratory information, self-reported medical and family history, physical activity patterns (Global Physical Activity Questionnaire (GPAQ)) [8], psychosocial stress, tobacco (WHO STEPwise surveillance questionnaire) [9] and alcohol use. Problematic alcohol use was assessed using the CAGE set of four questions [10]. Self-reported food intake during the preceding 24 hours was determined by a single 24-hour dietary recall using semi-structured interviews. Assets defining wealth were recorded and included ownership of consumer items (durable goods), dwelling characteristics in terms of wall and flooring materials, and the source of drinking water and toilet facilities. Psychosocial stress was examined using the following tools: 1) Brugha Life Events questionnaire (12 questions related to negative life events such as illness, death, financial or marital difficulties, etc. and their impact) [11], 2) Antonovsky's SOC (13 items measuring comprehensibility (cognitive), manageability (instrumental/behavioural) and meaningfulness (motivational)) [12], previously validated in South Africa [13], where a low SOC infers a low ability to cope with stressors [7], and 3) Locus of control (LOC) (six questions), which determines the individual's perceived sense of control over his/her environment and life with a low score construing poor perceived control and a high score good perceived control [14].

Three blood pressure (BP) measurements were taken at two-minute intervals using an Omron BP monitor with an appropriately sized cuff after the participant had been seated for five minutes. The average of the second and third BP measurements was used in the analysis. Height, weight, and waist and hip circumferences were measured using standardised techniques [15].

Blood samples, for glucose and lipid estimations, were drawn following an overnight fast of 10 hours, a standard oral glucose tolerance test (OGTT), using 75 grams of anhydrous glucose in 250 ml of water, was administered, and blood samples taken 120 minutes later [16]. Blood samples were kept on ice and transported to the laboratory within six hours to be centrifuged, aliquoted and stored at −80° until the assays were performed.

### Definitions

Diabetes, impaired glucose tolerance (IGT) and impaired fasting glucose (IFG) were diagnosed according to the 1998 WHO definition [16]. In addition, because the raw data for the initial study were unavailable for comparison, the 1985 WHO criteria [17], where the prevalences by age and gender were available, were used to compare the prevalence of diabetes in this study with that conducted in 1990.

Normal weight, overweight and obesity, raised waist circumference (>94 cm in men and >80 cm and in women), and raised waist-to-hip ratio (WHR) (>1.0 in men and >0.85 in women) were defined using standardised international criteria [18].

Hypertension was defined as BP≥140/90 mmHg for those without diabetes, and BP≥130/80 mmHg for those with diabetes, or using antihypertensive agents. Smoking status was defined as smoking ≥1 cigarette a day. Problematic alcohol use was deemed present if two or more CAGE questions were answered affirmatively [10]. Physical inactivity was defined as <150 minutes of moderate to vigorous activity per week. High fat intake was defined when >30% of total dietary intake was contributed by fat [19].

Dyslipidaemia was defined as follows [20]: total cholesterol (TC) >5 mmol/l, triglycerides >1.5 mmol/l, high density lipoprotein cholesterol (HDL-C) <1.2 mmol/l and calculated low density lipoprotein cholesterol (LDL-C) using the Friedewald equation >3.0 mmol/l [21] and HDL-C/TC ratio <20%.

### Statistical analysis

Data analyses were done using STATA 11 and SAS Version 9.2.3. Descriptive statistics, including crude prevalence, were calculated using the weights based on the sample design and adjusted for the realised sample. The dietary data was analysed using the Medical Research Council (MRC) Food Composition Tables and the MRC dietary analyses software Foodfinder. A principal component analysis of the pooled data, based on the assets that defined wealth, was used to develop an asset index [22]. Categories of relative wealth were created using tertiles. The age-standardised diabetes prevalence was calculated using the SEGI World Population as the standard [23]. Univariate analyses (sociodemographic and cardiovascular risk characteristics) are presented as mean values and standard deviations for continuous data, and as percentages for categorical data. For the pairwise comparison of normal glucose with IGT, unknown diabetes or known diabetes, a linear regression was done when the variables were continuous and a logistic regression when the variables were binary.

Survey multiple logistic regression analysis was used to determine the independent associations of diabetes with the psychosocial factors, adjusting for a set of modifiable (physical inactivity, fat intake, body mass index (BMI), waist and hip circumference) and non-modifiable (family history, age, sex and urbanisation) risk factors for diabetes as well as a socioeconomic confounder, housing type. Gender-specific models were done due to substantial differences in associations. To fully account for the known risk factors in the survey model, the non-linearity of their associations was investigated by means of generalised additive models (gam). To reflect the observed non-linearity, quadratic terms were added in the survey model for BMI and urbanisation in women. The waist and hip measurements, as well as their ratio, were highly correlated with BMI (r>0.85). Hence only BMI was retained in the models to avoid multicollinearity. The three psychosocial measures were modelled independently as continuous variables. SOC demonstrated a non-linear association with diabetes in women (significant cubic term) and cut-points were selected to mimic this pattern so as to enable statistical interpretation. Gender-specific cut-points were used for SOC and are indicated in the text and table.

The University of Cape Town's Research and Ethics Committee approved the study. All participants signed informed consent.

## Results

There were 1116 participants with 17 excluded because they did not meet the inclusion criteria. Of the 1099 participants included, 28 did not have blood glucose results available. The realised study sample comprised of 392 men and 707 women (64% and 108% of the planned sample, respectively). The response rate was 86% with 187 people selected who did not participate. Of the non-responders (i.e. the selected people who the study team were unsuccessful in contacting), 79 (42%) were men.

The crude and age-standardised prevalence of diabetes was 12.1% (95% confidence interval (CI): 10.2–14.0) and 13.1% (95%CI: 11.0–15.1), respectively, with higher rates found in women (crude: 13.8% (95%CI: 11.4–16.3); age-standardised: 14.7% (95%CI: 12.1–17.3)) compared to men (crude: 10.2% (95%CI: 7.1–13.4); age-standardised: 11.3% (95%CI: 8.0–14.6)). The prevalence of unknown diabetes was 4.9% with similar rates in men (4.8%) and women (4.9%). However, the rate of known diabetes (7.2%) was lower in men (5.4%) compared to women (8.9%).

**Figure 1:**
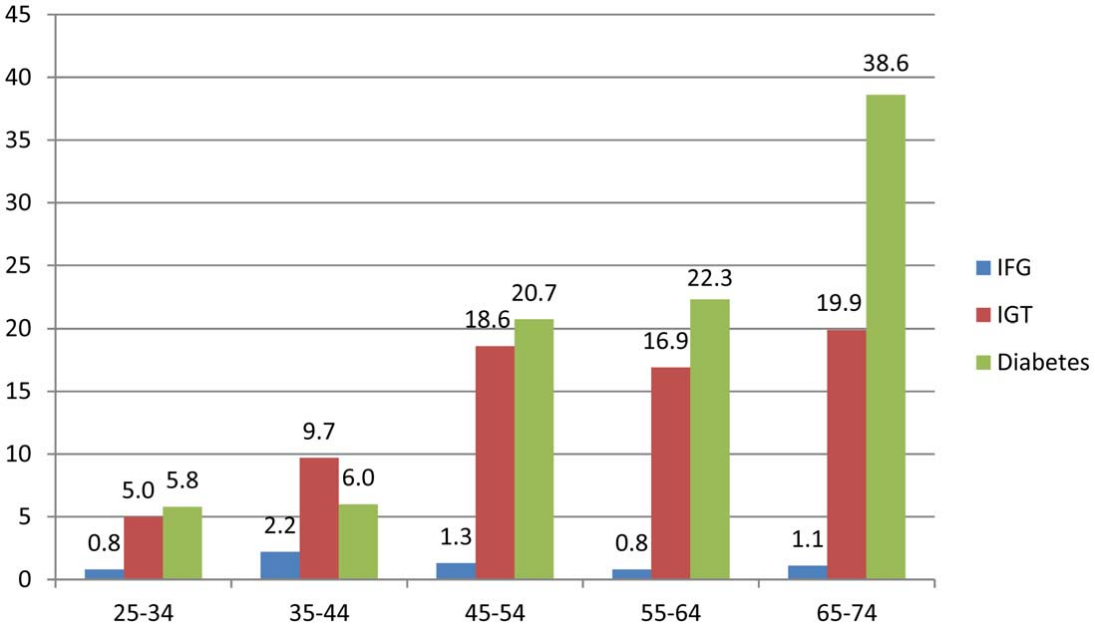
Prevalence of glycaemic categories based on 1998 WHO criteria presented by age categories. Footnote: IFG: impaired fasting glycaemia; IGT: impaired glucose tolerance.

The higher diabetes prevalence by age category is evident in Figure 1, peaking in 65–74-year-olds at 38.6%. The pattern by age category was similar for unknown and known diabetes with rates of 15.0% and 23.7%, respectively, in 65–74-year-old participants (data not shown).

The pattern for IGT was similar with rates >16% in those ≥45 years of age versus <10% in their younger counterparts. The crude and age-standardised prevalence of IGT was 10.7% (95%CI: 8.9–12.6) and 11.2% (95%CI: 9.2–13.1), respectively. The crude and age-standardised prevalence of IFG was 1.2% (95%CI: 0.6–1.9) and 1.2% (95%CI: 0.6–1.9).

**Figure 2:**
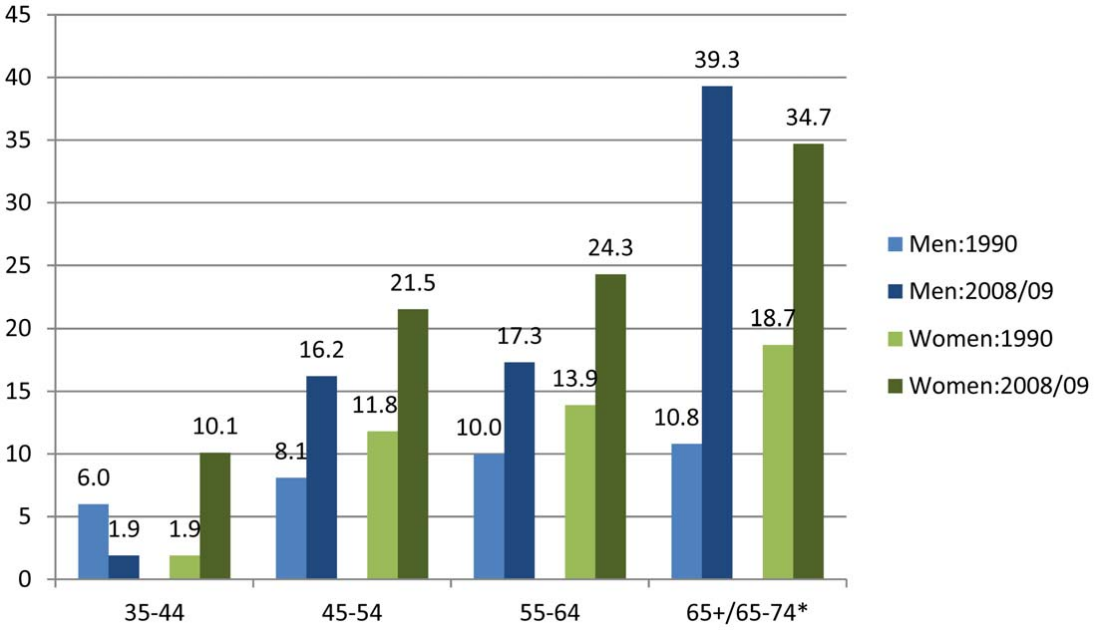
Diabetes prevalence based on 1985 WHO criteria presented by age categories for men and women in 1990 and 2008/09. Footnote: *1990: 65+ years, 2008/09: 65–74 years.

The age-standardised diabetes prevalence, by the 1985 WHO criteria, was 12.2% (95%CI: 10.2–14.2), higher than the 8.0% (95%CI: 5.8–10.3) prevalence found in 1990 in the same population in those >30 years of age [4]. The age-standardised IGT prevalence rose from 7.0% (95%CI: 4.9–9.1) in 1990 [4] to 11.7% (95%CI: 9.8–13.7) in 2008/09. Figure 2 demonstrates the markedly higher diabetes prevalence in 2008/09 compared to 1990 for men and women by all age categories except for men aged 35–44 years.

As seen in Table 1, participants with diabetes and IGT were older, had lower levels of education, and more were pensioners compared with the normoglycaemia group. Significantly fewer participants with known, but not unknown, diabetes lived in informal shacks, compared to the normoglycaemia group. However, only participants with unknown diabetes were significantly wealthier than those with normoglycaemia. Participants with known diabetes perceived their health to be poor, had significantly lower SOC and LOC scores, and were more affected by adverse life events that had occurred >6 months ago, compared to those with normoglycaemia.

**Table 1:** Socio-demographic and psychosocial factors presented by glycaemic categories based on 1998 WHO criteria (n = 1071)^a^.

	Total	Category of glycaemia					
		Normal	IFG	IGT	Diabetes		
					All	Unknown	Known
Number	1071	772	14	125	160	59	101
Age in years, mean ±SD	43.3±12.8	40.8±12.0	42.5±12.3	48.8±11.9***	51.8±12.3	51.7±12.6***	51.9±12.3***
***Socio-demographic factors, %***							
Gender, %							
Men	47.5	49.4	53.1	47.9	40.1	47.0	35.4*
Women	52.5	50.6	46.9	52.1	59.9	53.0	64.6*
Education, %:							
≤7 years	34.1	30.1	36.2	43.8**	47.1	45.3*	48.4**
>7 years	65.9	69.9	63.8	56.2**	52.9	54.7*	51.7**
Employment Status, %:							
Employed	22.8	24.5	21.0	17.7	15.3	9.1*	19.4
Unemployed	61.0	63.7	74.4	56.8	53.6	63.6	46.8**
Pensioner	10.1	5.5	4.6	21.1***	27.6	25.5***	29.0***
Other	16.2	6.3	0.0	4.4	3.5	1.8	4.8
Housing Type, %:							
Built formal unit (private)	22.0	20.5	33.0	16.5	34.9	34.2*	35.3**
Council/core house/hostel	26.6	25.6	24.8	24.7	33.0	24.6	38.7**
Informal shack/other	51.4	53.9	42.2	58.8	32.1	41.2	26.0***
Occupancy rate(persons/room), mean±SD	2.5±1.6	2.5±1.6	2.5±1.8	2.3±1.5	2.2±1.5	2.1±1.5*	2.3±1.5
% life in urban area, mean ±SD	61.9±32.6	60.4±33.7	60.3±31.6	64.1±30.0	67.8±29.1	64.9±30.3	69.6±28.4**
Asset Index by tertiles, %:							
1^st^ (poorest)	33.2	34.4	28.4	33.7	26.4	28.2	25.2
2^nd^	33.7	33.8	44.1	35.1	29.2	19.0*	36.1
3^rd^ (richest)	33.1	31.9	27.4	31.3	44.4	52.8**	38.7
***Psychosocial factors/influences, %***							
Perceived health status:							
Poor	19.5	17.7	10.5	19.5	31.5	20.7	38.8***
Average	53.7	53.5	55.1	57.6	52.6	50.0	54.3
Good to excellent	26.8	28.8	34.4	22.9	15.9	29.3	6.8***
Living with HIV/AIDS in the household	20.9	20.1	20.2	16.0	26.9	27.0	26.8
Sense of coherence: score mean ±SD	54.5±10.4	54.4±10.4	63.4±10.0	55.2±10.3	53.7±10.4	56.4±10.8	52.1±9.8*
Locus of control: score mean ±SD	18.7±3.0	18.8±3.0	20.4±2.4	18.4±2.9	18.6±3.1	19.6±3.2	18.1±2.8*
Total Life events: score mean ±SD	7.6±2.9	7.6±2.8	6.1±2.1	7.5±3.0	7.6±2.9	7.1±3.2	7.9±2.7
Impact within last 6 months	3.9±2.9	4.1±2.9	3.6±2.56	3.8±2.9	3.6±2.9	3.2±3.2*	3.8±2.7
Lifetime impact >6 months	9.9±4.3	9.8±4.3	7.4±3.13	10.0±4.6	10.5±4.2	9.6±4.1	11.0±4.2*

a : Of the total sample of 1099 participants, 28 did not have blood results; IFG: impaired fasting glycaemia; IGT: impaired glucose tolerance; Diabetes: WHO definition or using hypoglycaemic agents or told by doctor/nurse; Mean ±SD are reported for the study sample and not adjusted for the population; Other: homemakers, students and those receiving disability grants; For pairwise comparison of normal glucose with: IGT, unknown diabetes or known diabetes:

* <0.05;

** <0.01;

*** <0.001.

The mean values of the risk factors, with the exception of HDL-C, were generally significantly worse in participants with known and unknown diabetes compared to those with normoglycaemia (Table 2). The prevalence of CVD risk factors was higher within glycaemic categories from normoglycaemia to IGT to diabetes as illustrated in Table 3. Participants with known and unknown diabetes, compared to those with normoglycaemia, had significantly higher rates of hypertension, hyperlipidaemia and raised adiposity. However, known, but not unknown, diabetes was significantly associated with a family history of diabetes and lower rates of smoking compared to normoglycaemia. Participants with unknown diabetes had significantly higher rates of low physical activity levels than the normoglycaemic group.

**Table 2:** Means and standard deviations (±SD) of cardiovascular disease risk factors presented by glycaemic categories based on 1998 WHO criteria (n = 1071)^a^.

	Total	Category of glycaemia					
		Normal	IFG	IGT	Diabetes		
mean, SD					All	Unknown	Known
Number	1071	772	14	125	160	59	101
***Plasma glucose:*** (mmol/l)							
Fasting glucose	5.4±2.5	4.6±0.7	6.5±0.3	5.3±0.7***	9.3±4.8	8.2±3.4***	10.0±5.3***
2-hr glucose	6.9±4.2	5.3±1.3	5.8±1.4	8.9±0.9***	14.3±7.2	13.5±5.0***	14.9±8.4***
***Lipids:*** (mmol/l)							
Total cholesterol	4.4 ±1.12	4.3±1.1	4.5±1.6	4.7±1.2***	4.8±1.31	4.9±1.2***	4.7±1.4***
HDL-C	1.17±0.5	1.17±0.4	1.40±0.7	1.25±0.6	1.13±0.4	1.17±0.4	1.10±0.4
LDL-C	3.0±1.0	2.9±0.9	2.8±1.4	3.2±1.0**	3.4±1.1	3.4±1.1***	3.4±1.1***
Triglycerides	1.1±0.9	1.0±0.8	1.2±0.5	1.3±0.8***	1.6±1.3	1.4±0.7**	1.7±1.5***
HDL-C:TC, %	27.2±9.9	27.8±9.1	37.9±35.2	26.6±9.9	24.5±8.2	24.7±8.2*	24.3±8.3**
***Blood pressure (BP):*** (mmHg)							
Systolic BP	126.0±23.3	123.3±22.4	126.8±14.9	130.8±22.4**	136.6±25.9	137.6±25.3***	135.6±26.4***
Diastolic BP	82.0±13.3	80.8±13.3	81.8±10.8	84.8±12.5**	85.8±13.0	87.4±13.1***	84.9±12.9**
***Anthropometry:***							
BMI (kg/m^2^)	29.9±8.5	28.8±8.1	27.2±5.3	31.2±9.5**	33.4±8.0	33.9±8.7***	33.2±7.5***
Waist circumference (cm)	93.3±15.6	90.8±14.9	90.7±10.0	96.2±16.9***	102.2±14.9	102.7±15.4***	101.8±14.6***
Waist-to-hip ratio	0.87±0.1	0.85±0.1	0.89±0.1	0.88±0.1***	0.91±0.1	0.91±0.1***	0.90±0.1***

a : Of the total sample of 1099 participants, 28 did not have blood results; IFG: impaired fasting glycaemia; IGT: impaired glucose tolerance; Diabetes: WHO definition or using hypoglycaemic agents or told by doctor/nurse; Mean ±SD are reported for the study sample and not adjusted for the population; HDL-C: high density lipoprotein cholesterol; LDL-C: low density lipoprotein cholesterol; TC: total cholesterol; BMI: body mass index; For pairwise comparison of normal glucose with: IGT, unknown diabetes or known diabetes:

* <0.05;

** <0.01;

*** <0.001.

**Table 3:** Prevalence of cardiovascular disease and risk factors presented by glycaemic categories based on 1998 WHO criteria (n = 1071)^a^.

	Total	Category of glycaemia					
		Normal	IFG	IGT	Diabetes		
					All	Unknown	Known
Number	1071	772	14	125	160	59	101
***Personal medical history of: (%)***							
Ischaemic heart disease	3.0	2.0	0.0	2.0	8.4	8.7**	8.1**
Stroke	3.4	2.8	0.0	1.9	7.7	7.1	8.1*
***Family history of: (%)***							
Diabetes	22.2	19.8	10.5	23.5	36.8	22.8	46.2***
***Hypertension: (%)***							
BP≥130/80 mmHg or using antihypertensive agents	57.0	51.4	45.7	69.1**	82.6	83.3**	82.1***
BP≥140/90 mmHg or using antihypertensive agents	37.5	30.2	35.8	55.6***	65.9	56.9***	72.0***
***Dyslipidaemia: (%)***							
Total cholesterol >5 mmol/l	24.1	19.7	34.1	37.7***	37.3	34.7**	39.1***
HDL-C <1.2 mmol/l	61.3	60.9	55.9	60.6	68.1	69.9	66.8
LDL-C>3 mmol/l	42.0	37.1	49.6	55.9***	59.0	60.4**	58.1**
Triglycerides >1.5 mmol/l	16.2	11.3	35.2	30.4***	32.0	30.6***	32.9***
HDL-C:TC <20%	16.8	13.1	13.1	26.8**	30.3	32.3**	29.0**
***Anthropometry: (%)***							
BMI≥25 kg/m^2^	57.2	52.3	57.9	62.3	81.0	72.3*	86.9***
Raised waist circumference	54.8	48.7	72.1	60.8*	80.4	76.6**	82.9***
Raised waist-to-hip ratio	29.4	23.7	29.9	42.6***	48.2	39.3*	54.2***
***Lifestyle/behavioural risk factor: (%)***							
Smoke: ≥1cigarette/day,	27.6	30.2	44.1	28.6	11.9	18.8	7.3***
Problematic alcohol use: CAGE ≥2	33.1	36.3	39.4	31.4	15.7	16.0**	15.4***
Moderate to vigorous activity/week: <150 minutes	6.6	6.0	10.5	8.4	8.6	17.1**	2.9
***Diet: (%)***							
Fat intake ≥30% of diet	36.1	37.0	28.9	35.4	29.7	29.8	29.7

a : Of the total sample of 1099 participants, 28 did not have blood results; IFG: impaired fasting glycaemia; IGT: impaired glucose tolerance; Diabetes: WHO definition or using hypoglycaemic agents or told by doctor/nurse; HDL-C: high density lipoprotein cholesterol; LDL-C: low density lipoprotein cholesterol; TC: total cholesterol; BMI: body mass index; Raised waist circumference: men >94 cm, women >80 cm; Raised waist-to-hip ratio: men >1.0, women >0.85; For pairwise comparison of normal glucose with: IGT, unknown diabetes or known diabetes:

* <0.05;

** <0.01;

*** <0.001.

Among participants with diabetes, 57.9% (men: 48.2%. women: 64.4%) were aware of their condition and 38.6% (men: 31.3%, women: 43.5%) were on treatment. Of the latter, 25.5% (men: 22.6%, women: 26.9%) and 30.8% (men: 29.8%, women: 31.3%) had fasting glucose levels <6.0 mmol/l and <8.0 mmol/l, respectively.

In the multiple logistic models for women, where the three psychosocial measures were entered independently, only SOC was significantly associated with diabetes (Table 4). Women with SOC scores ≤40 had an increased risk of diabetes (odds ratio (OR): 2.57, 95%CI: 1.37–4.80, p = 0.003). In the model with LOC, the odds for diabetes in women with higher LOC scores was 1.03, 95%CI: 0.95–1.12, p = 0.450. The model with adverse life events showed that the odds for diabetes with increasing adverse life events was 1.00, 95%CI: 0.96–1.03, p = 0.857 (data not shown). The other variables in the model with SOC for women that were significantly associated with diabetes included older age (OR: 1.05, 95%CI: 1.03–1.08, p<0.001), family history of diabetes (OR: 3.13, 95%CI: 1.92–5.12, p<0.001), higher BMI levels (OR: 1.48, 95%CI: 1.20–1.82, p<0.001) reaching a plateau after 30 kg/m^2^, and living in built formal housing compared to informal shacks (OR: 1.75, 95%CI: 1.01–3.04, p = 0.047). Physical inactivity, high fat intake and urbanisation failed to achieve significance in the regression model for women. When waist circumference replaced BMI in the same model, the odds for diabetes with increasing waist circumference, after adjusting for the other factors, was significant (OR: 1.29, 95%CI: 1.09–1.51, p = 0.003). There was no change in the significance or direction of the other variables in this model.

**Table 4:** Multiple logistic regression model for associations with diabetes in women and men**.**

Variables	Women				Men			
	Odds Ratio	95% Confidence Interval		p-value	Odds Ratio	95% Confidence Interval		p-value
		Lower limit	Upper limit			Lower limit	Upper limit	
Age (years)	1.05	1.03	1.08	**<0.001**	1.05	1.02	1.08	**0.002**
Family history of diabetes (yes)	3.13	1.92	5.12	**<0.001**	1.09	0.51	2.32	0.829
BMI: linear (weight in kg/height in m^2^)	1.48	1.20	1.82	**<0.001**	1.10	1.04	1.18	**0.003**
quadratic (weight in kg/height in m^2^)^2^	0.995	0.992	0.998	**0.001**	NA	-	-	**-**
Moderate to vigorous physical activity(<150 min/week)	1.99	0.90	4.42	0.090	NA	-	-	**-**
Moderate to vigorous physical activity (min/week)	N/A	-	-	**-**	1.00	0.98	1.02	0.734
Dietary fat intake (%)	1.01	0.99	1.03	0.554	0.99	0.97	1.01	0.403
Urbanisation: linear (% of life in city)	1.04	0.98	1.10	0.111	1.00	0.99	1.01	0.552
quadratic (% of life in city)^2^	1.00	0.99	1.00	0.130	N/A	-	-	**-**
Housing Type: Informal shack/other	1.00				1.00			
Council/core house/hostel	1.56	0.86	2.86	0.144	2.28	0.98	5.33	0.057
Built formal unit (private)	1.75	1.01	3.04	**0.047**	1.24	0.54	2.86	0.613
Sense of coherence score: scored ≤40	2.57	1.37	4.80	**0.003**	NA	-	-	-
scored ≤68	N/A	-	-	**-**	2.18	0.49	9.64	0.301

N/A: this factor is not applicable for the gender specific model. Men: quadratic terms for BMI and urbanisation not used in the model. Physical activity used as a continuous variable in men because too few had <150 minutes/week of activity. Gender-specific cut-points used for sense of coherence score.

In the models for men, none of the psychosocial measures were significantly associated with diabetes. The association of the psychosocial scores with diabetes in the three models were as follows: SOC≤68: OR: 2.18, 95%CI: 0.49–9.64, p = 0.301; higher LOC: OR: 1.07, 95%CI: 0.93–1.22, p = 0.354; and increasing adverse life events: OR: 1.02, 95%CI: 0.98–1.06, p = 0.448. Of the other risk factors in the model with SOC, only older age (OR: 1.05, 95%CI: 1.02–1.08, p = 0.002) and higher BMI (OR: 1.10, 95%CI: 1.04–1.18, p = 0.003) were significantly associated with diabetes (Table 4). When waist circumference replaced BMI in the SOC model, the risk for diabetes, after adjusting for the other factors, increased 4.0% for every centimetre around the waist (OR: 1.04, 95%CI: 1.01–1.07, p = 0.004). There was no change in the significance or direction of the other variables in this model.

## Discussion

This study is the first in Southern Africa to demonstrate substantially higher diabetes prevalence in urban black African residents compared to two decades ago. Furthermore, the age-standardised diabetes prevalence is among the highest reported in SSA; the 12.2% prevalence with a high degree of uncertainty (95% CI: 5.4–23.2) found in a small opportunity sample (n = 281) of 17–68 year old urban Kenyans [24] is not directly comparable. In 1990 too, the prevalence of diabetes was considerably higher than that reported from elsewhere in SSA, which was thought to be primarily due to the higher prevalence of overweight/obesity in the Cape Town population [2], [4].

Another notable finding is that the prevalence of diabetes showed a steep rise in people over the age of 45 years, typical of developing countries, with 20–25% of participants between the economically-active years of 45–64 screening positive for diabetes. However, the peak diabetes prevalence in this study was in 65–74-year-old participants, similar to developed countries where diabetes occurs predominantly in those ≥65 years of age [25]. Nonetheless, in Cameroon, dysglycaemia in women by age group was also reported to be highest in those 65 years and older, while there was no significant difference in men by age [26].

The low diabetes prevalence in 35–44-year-old men (1.9%) was unexpected and an explanation is not readily apparent. It is unlikely to be related to a small number of participants in this age group as they numbered over 100. It is however conceivable that this may be a survivor effect, in that those who had AIDS at an earlier age have died.

The prevalence of IGT followed a similar age trend with markedly higher rates in those over 45 years (18.4%) compared to younger participants (6.6%). The overall IGT prevalence was amongst the highest found in SSA where this has generally been reported to be <10% [1]. Considering that up to 70% of those with IGT may progress to diabetes [24], the markedly higher IGT in 2008/09 compared to 1990 may indicate a dramatic increase in future diabetes in this population.

The low prevalence of IFG in this study was unexpected, but it does underscore the need for the OGTT in epidemiological studies to identify those with IGT when estimating future trends in diabetes. IGT and IFG are not interchangeable and denote different abnormalities of glucose regulation [16]. Furthermore, only 16 (27.1%) of the newly diagnosed diabetic participants (n = 59) in this study were diagnosed on fasting glucose levels. This is in keeping with previous studies from SSA in which diabetes prevalence rates were lower when based on only fasting glucose compared to the OGTT [1], [27].

The high prevalence of diabetes and IGT in this study may be attributed to the high rate of some diabetes risk factors. One of these is obesity. Raised adiposity, the most potent of the risk factors [5], was present in ≥50% of the total study sample, and in over 80% of participants with diabetes when measured by BMI ≥25 kg/m^2^. Centralisation of fat, as evidenced by waist circumference and WHR were also common in the whole sample and higher in those with diabetes. Of note is that both increasing BMI and waist circumference were associated with diabetes in the regression analyses for men and women.

The lack of an overall significant association between low physical activity levels and diabetes in the present study was unexpected, and may reflect over-reporting of activity, particularly among participants with known diabetes. Known compared to unknown diabetic participants had much lower rates of inadequate physical activity levels (2.9% vs. 17.1%). Women with diabetes had higher rates of inadequate physical activity (11.6%) compared to women without diabetes (6.1%) (p = 0.035) but there was no difference in men (diabetes: 4.1% vs. no diabetes: 6.5% p = 0.467). These results highlight the need for an objective measure of physical activity.

Urbanisation was associated with known, but not unknown, diabetes in the univariate analysis but not with diabetes overall in the regression model. It is likely to be due to the ‘bluntness’ of the proxy we used for urbanisation i.e. proportion of life spent in the city. It may also reflect the rapidity with which new migrants to the city adopt unhealthy lifestyle behaviours that predispose to diabetes.

A low SOC was significantly associated with diabetes in women in this study, similar to that reported in 35–56-year-old women in Sweden [7]. Although no association was found between low SOC and diabetes in men, possibly due to the small number of men with diabetes (n = 45), a cohort study in Finland reported a 46% higher risk of diabetes in men with low SOC aged ≤50 years of age on entry into the study [28]. As this is a cross-sectional study, no causal relationship can be assumed between SOC and diabetes in women. This is particularly pertinent because the univariate analyses showed that SOC was significantly lower in known, but not unknown, diabetes compared with normoglycaemia. This suggests that stress imposed by diabetes may contribute to a lower SOC. The association of SOC with the development of diabetes in this setting needs to be examined in longitudinal studies.

Of interest is that two of the 12 adverse life events examined were significantly associated with diabetes in both men and women in the univariate analyses. These were death of a parent, child or spouse, and separation caused by marital difficulties.

The minimal improvement in the proportion of participants with a prior diagnosis of diabetes compared to the 1990 study (57.9% vs. 52.2%) [4], indicates that screening for common chronic non-communicable diseases such as diabetes may not be receiving the necessary attention. This is likely to be due to primary healthcare facilities being overwhelmed by the multiple disease burdens experienced by South Africans, in particular HIV/AIDS.

Nonetheless, timeous diagnosis and treatment is essential; late diagnosis often has serious and fatal consequences such as early presentation of diabetic complications and premature mortality [1]. Comprehensive diabetes care that includes attention given to the other CVD risk factors needs to be a key strategy considering that the cost of optimal treatment is relatively low compared to the management of diabetes complications.

The limitations of this study include: 1) the cross-sectional design which precludes any causal association between diabetes and SOC, 2) the low sample realisation in men (64%), which is characteristic of epidemiological studies in this country, probably due to their reluctance to participate, particularly for the drawing of blood samples, which necessitated higher sampling weights and loss of precision, 3) a single 24-hour dietary recall of the previous day's food intake which may account for the lack of significance between total fat intake and diabetes, and 3) the use of self-reported rather than objectively measured ambulation or physical activity.

## Conclusion

The current higher prevalence of diabetes in the urban black African population of Cape Town compared to two decades ago is of considerable concern. Indeed the burden of diabetes in this setting can be predicted to increase further in view of the high rates of IGT and the extremely high levels of overweight/obesity. Concerted measures to raise public awareness of modifiable diabetes risk factors and healthier lifestyles are needed with policies implemented to facilitate behaviour change. Targeted screening programmes should be considered to address the suboptimal detection of diabetes. Finally, the importance of social support cannot be overlooked in developing diabetes prevention strategies. If longitudinal studies find SOC to be involved in the development of diabetes in the African setting, diabetes prevention strategies should also incorporate measures to modify this risk.

## References

[1] Mbanya JC, Motala AA, Sobngwi E, Assah FK, Enoru ST (2010) Diabetes in sub-Saharan Africa. Lancet 375: 2254–2266. 20609971 10.1016/S0140-6736(10)60550-8

[2] Levitt NS (2008) Diabetes in Africa: epidemiology, management and healthcare challenges. Heart 94: 1376–1382. 18519551 10.1136/hrt.2008.147306

[3] Bradshaw D, Norman R, Pieterse D, Levitt NS (2007) Estimating the burden of disease attributable to diabetes in South Africa in 2000. S Afr Med J 97: 700–706. 17952227

[4] Levitt NS, Katzenellenbogen JM, Bradshaw D, Hoffman MN, Bonnici F (1993) The prevalence and identification of risk factors for NIDDM in urban Africans in Cape Town, South Africa. Diabetes Care 16: 601–607. 8462387 10.2337/diacare.16.4.601

[5] Mensah GA, Mokdad AH, Ford E, Narayan KM, Giles WH, et al (2004) Obesity, metabolic syndrome, and type 2 diabetes: emerging epidemics and their cardiovascular implications. Cardiol Clin 22: 485–504. 15501618 10.1016/j.ccl.2004.06.005

[6] Mooy JM, de Vries H, Grootenhuis PA, Bouter LM, Heine RJ (2000) Major stressful life events in relation to prevalence of undetected type 2 diabetes: the Hoorn Study. Diabetes Care 23: 197–201. 10868831 10.2337/diacare.23.2.197

[7] Agardh EE, Ahlbom A, Andersson T, Efendic S, Grill V, et al (2003) Work stress and low sense of coherence is associated with type 2 diabetes in middle-aged Swedish women. Diabetes Care 26: 719–724. 12610028 10.2337/diacare.26.3.719

[8] World Health Organization, Department of Noncommunicable Diseases (2002) Global Physical Activity Questionnaire and Analysis Guide.

[9] Bonita R, deCourten M, Dwyer T, Jamrozik K, Winkelmann R (2002) Surveillance of risk factors for non-communicable disease: The WHO STEP-wise Approach Geneva: World Health Org.

[10] Ewing JA (1984) Detecting alcoholism. The CAGE questionnaire. JAMA 252: 1905–1907. 6471323 10.1001/jama.252.14.1905

[11] Brugha T, Bebbington P, Tennant C, Hurry J (1985) The List of Threatening Experiences: a subset of 12 life event categories with considerable long-term contextual threat. Psychol Med 15: 189–194. 3991833 10.1017/s003329170002105x

[12] Eriksson M, Lindstrom B (2005) Validity of Antonovsky's sense of coherence scale: a systematic review. J Epidemiol Community Health 59: 460–466. 15911640 10.1136/jech.2003.018085 PMC1757043

[13] Botha KF, Du Plessis WF, Van Rooyen JM, Wissing MP (2002) Biopsychosocial determinants of self-management in culturally diverse South african patients with essential hypertension. J Health Psychol 7: 519–531. 22113138 10.1177/1359105302007005672

[14] Rosengren A, Hawken S, Ounpuu S, Sliwa K, Zubaid M, et al (2004) Association of psychosocial risk factors with risk of acute myocardial infarction in 11119 cases and 13648 controls from 52 countries (the INTERHEART study): case-control study. Lancet 364: 953–962. 15364186 10.1016/S0140-6736(04)17019-0

[15] Alberti KG, Zimmet P, Shaw J (2006) Metabolic syndrome–a new world-wide definition. A Consensus Statement from the International Diabetes Federation. Diabet Med 23: 469–480. 16681555 10.1111/j.1464-5491.2006.01858.x

[16] World Health Organization (1999) Definition, Diagnosis and Classification of Diabetes Mellitus and its Complications: Report of a WHO Consultation. Geneva: World Health Org.

[17] World Health Organization (1985) Diabetes Mellitus: Report of a WHO Study Group. Geneva: World Health Org. 3934850

[18] World Health Organization (2000) Obesity: Preventing and Managing the Global Epidemic: Report of a WHO Consultation. Geneva: World Health Org. 11234459

[19] Joint WHO/FAO Expert Consultation (2003) Diet, Nutrition and the Prevention of Chronic Diseases. Geneva: World Health Org.

[20] South African Medical Association and Lipid and Atherosclerosis Society of Southern Africa Working Group (2000) Diagnosis, management and prevention of the common dyslipidaemias in South Africa–clinical guideline, 2000. S Afr Med J 90: 164–178, 164-174, 176-179. 10745972

[21] Friedewald WT, Levy RI, Fredrickson DS (1972) Estimation of the concentration of low-density lipoprotein cholesterol in plasma, without use of the preparative ultracentrifuge. Clin Chem 18: 499–502. 4337382

[22] Filmer D, Pritchett LH (2001) Estimating wealth effects without expenditure data–or tears: an application to educational enrollments in states of India. Demography 38: 115–132. 11227840 10.1353/dem.2001.0003

[23] Doll R, Smith P (1982) Comparison between registries: age-standardized rates In: Waterhouse J, Muir C, Shanmugaratnam K, editors. Cancer Incidence in Five Continents. Lyon, France: IARC Scientific Publications. pp. 671–675. 1284608

[24] Christensen DL, Friis H, Mwaniki DL, Kilonzo B, Tetens I, et al (2009) Prevalence of glucose intolerance and associated risk factors in rural and urban populations of different ethnic groups in Kenya. Diabetes Res Clin Pract 84: 303–310. 19361878 10.1016/j.diabres.2009.03.007

[25] King H, Aubert RE, Herman WH (1998) Global burden of diabetes, 1995–2025: prevalence, numerical estimates, and projections. Diabetes Care 21: 1414–1431. 9727886 10.2337/diacare.21.9.1414

[26] Sobngwi E, Mbanya JC, Unwin NC, Porcher R, Kengne AP, et al (2004) Exposure over the life course to an urban environment and its relation with obesity, diabetes, and hypertension in rural and urban Cameroon. Int J Epidemiol 33: 769–776. 15166209 10.1093/ije/dyh044

[27] Motala AA, Esterhuizen T, Gouws E, Pirie FJ, Omar MA (2008) Diabetes and other disorders of glycemia in a rural South African community: prevalence and associated risk factors. Diabetes Care 31: 1783–1788. 18523142 10.2337/dc08-0212 PMC2518345

[28] Kouvonen AM, Vaananen A, Woods SA, Heponiemi T, Koskinen A, et al (2008) Sense of coherence and diabetes: a prospective occupational cohort study. BMC Public Health 8: 46. 18254945 10.1186/1471-2458-8-46 PMC2268681

